# The association between plant-based diet and erectile dysfunction in Chinese men

**DOI:** 10.1186/s12610-021-00129-5

**Published:** 2021-05-13

**Authors:** Yi Lu, Jiaqi Kang, Zhongjia Li, Xiao Wang, Kang Liu, Kechong Zhou, Wei Wang, Chen Shen

**Affiliations:** 1 Department of Urology, The Second Affiliated Hospital of Dalian Medical University, 467 Zhongshan Road, Shahekou District, Dalian, 116023 China; 2 Tianjin Medical University, Tianjin, 300070 China; 3 Department of Urology, Tianjin Medical University General Hospital, Tianjin, 300052 China

**Keywords:** Régime, Régime à base de Végétaux, Dysfonction érectile, Fonction érectile, Testostérone, Diet, Plant-based diet, Erectile dysfunction, Erectile function, Testosterone

## Abstract

**Background:**

Diet, one of the components of lifestyle, has been believed to have associations with erectile dysfunction (ED). However, whether there is an association between plant-based diet and ED is remains to be explored. Thus, we conducted the nested case-control study to investigate the relationship between the plant-based diet and ED in China.

**Results:**

ED group (92 subjeczts) and ED free group (92 subjects) were similar in terms of basic features (*P* > 0.05), except for lifestyle (*P* < 0.05). The plant-diet index (PDI) and healthy plant-diet index (hPDI) in the ED group were significantly lower than those in the control group (*P* < 0.001). Adjusted multivariate analysis indicated that the presence of ED was negatively associated with nitric oxide levels, PDI, and hPDI (all *P* < 0.05), and was positively related to body mass index, metabolic syndrome, and E-selectin levels. Furthermore, both the PDI and hPDI increased significantly as the International Index of Erectile Function (IIEF-5) scores increased within the ED group (*P* < 0.05). Multi-model multivariate analysis indicated the robustness of results.

**Conclusions:**

More plant-based diet intake was associated with a reduced presence of ED and less severe ED in China. Committing to plant-based diet can be encouraged for many health benefits and to lower ED burden. Further well-designed studies are warranted to validate our findings.

**Supplementary Information:**

The online version contains supplementary material available at 10.1186/s12610-021-00129-5.

## Résumé

**Contexte:**

L’alimentation, l’une des composantes du mode de vie, a été soupçonnée d’avoir des associations avec la dysfonction érectile (DE). Toutefois, il reste à explorer s’il existe une association entre l’alimentation à base de plantes et la DE. Ainsi, avons-nous mené une étude cas-témoins imbriquée pour étudier la relation entre le régime à base de plantes et la DE en Chine.

**Résultats:**

Le groupe avec DE (92 sujets) et le groupe sans DE (92 sujets) étaient semblables en termes de caractéristiques de base (*p* > 0.05), à l’exception du mode de vie (*p* < 0.05). L’indice de régime végétal (IRV) et l’indice de régime végétal sain (IRVS) dans le groupe avec DE étaient sensiblement inférieurs à ceux du groupe témoin (*p* < 0.001). L’analyse multivariée ajustée a indiqué que la présence de la DE était négativement associée aux niveaux d’oxyde nitrique, de IRV, et de IRVS (tous *p* < 0.05), et était positivement liée à l’indice de masse corporelle, au syndrome métabolique, et aux niveaux d’E-sélectine. En outre, l’IRV et l’IRVS ont augmenté de façon significative à mesure que les scores de l’Indice international de fonction érectile (IIEF-5) augmentaient au sein du groupe avec DE (*p* < 0,05). L’analyse multivarié multi-modèles a indiqué la robustesse des résultats.

**Conclusions:**

Un apport alimentaire plus riche en végétaux était associé à une présence réduite de la DE et à une DE moins grave, en Chine. S’engager dans un régime alimentaire à base de végétaux peut être propice à de nombreux avantages pour la santé et à la réduction du fardeau constitué par la DE. D’autres études bien conçues sont justifiées pour valider nos résultats.

## Background

Diet modification, proved by several well-designed studies, is considered as a potentially important method for cancer prevention and chronic non-communicable diseases control [1–3]. In light of these findings, participation in diet modification, especially the plant-based diet has grown swiftly in recent years. In the United States, the number of plant-based diet consumers was reported to increase 5 times between 2014 and 2017 and sales of the diet has also increased by 20% between 2017 and 2018 [4]. Plant-based diet helps to transform diet habits from consumption of fish, dairy, meats, and poultry to the intake of plant foods [5]. The promotion of plant-based diet can benefit both public health and the environment. It was indicated that such a diet could reduce the incidence of metabolic syndrome (MetS), heart disease, cancer, and even mortality [6–8]. Furthermore, it was demonstrated that a suitable reduction of animal-based diet and a more sustainable plant-based diet pattern reduced about 70% of the greenhouse gas emissions [9].

A normal penile erection has always been considered as the symbol of a man’s virility and sexual ability. Complex mechanisms, involving the interplay between vascular and neurological events exist in the erectile process. Recently, the vital role of nitric oxide (NO) in the relaxation and erection of penile smooth muscle has been widely accepted [10]. Erectile function (EF) is a multidimensional process, any alteration in sexual response, organic, relational, and emotional will lead to erectile dysfunction (ED). ED is defined as a persistent, or occasional, difficulty in achieving or maintaining an erection status sufficient for satisfactory sexual performance [11]. Risky factors of ED involved several lifestyle factors, including smoking, excessive alcohol intake, lack of physical exercise, and unhealthy diets. In recent years, it has been indicated that Mediterranean diet and pistachio consumption can improve EF by increasing antioxidants, and arginine, which is the precursor of NO that can increase vasodilatation [12]. However, it is still unclear whether there is an association between plant-based diet and ED. Additionally, most studies of plant-based diet include no quantitative data, which makes it difficult to exactly grade the intake of vegetarian diets [13]. In line with this, we aimed to report the plant-based diet status in a Chinese male population and to investigate the association between the diet and ED presence and ED severity by using a graded method.

## Material and methods

### Subjects

**Fig. 1:**
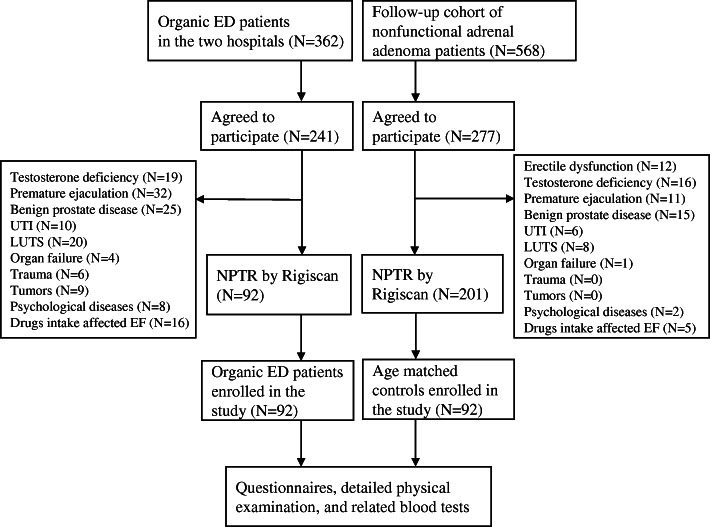
Flowchart of the subject selection. Abbreviations: ED: erectile dysfunction; EF: erectile function. LUTS: lower urinary tract symptoms; UTI: urinary tract infection

Between February 2019 and May 2020, we selected 92 patients who were diagnosed as organic ED and 92 age-matched male controls in Tianjin. Individuals without ED in the control group were selected from patients with nonfunctional adrenal adenoma who applied for follow-up. Subjects who had testosterone deficiency, premature ejaculation (PE), prostate disease, including prostatitis and benign prostatic hyperplasia, urinary tract infection, lower urinary tract symptoms, any organ failure, pelvic or perineal trauma, any malignancies, and any psychological/neurological/psychiatric disorders were all excluded. Subjects who take drugs that could affect the sexual function were also excluded. All the subjects had normal pubertal development. All the ED patients were firstly turned to andrologists for their erectile problems and all of them had been in a stable and heterosexual relationship with the same partner for at least 6 months. Before the enrollment, each of the patients complaining of ED went through nocturnal penile tumescence and rigidity (NPTR) monitoring by Rigiscan (Timm Medical Technologies, GOTOP, USA) for three separate nights to objectively differentiate organic and psychogenic ED. A flowchart of the selection process was shown in Fig. 1.

### Study design and procedures

Ethics approval was obtained on December 22, 2018 from hospital ethics committee. We asked each patient to provide written informed consent before data collection and provided a summary of the project to each of them to introduce the aim and details of our research.

The study was composed of 3 parts: self-completion questionnaires, detailed physical examination, and related blood tests. The questionnaire piloted in Simplified Chinese (Mandarin) included the following items: (I) Basic features (e.g. age, partner age, duration of partnership, etc.); (II) Sexual and medical history; (III) International Index of Erectile Function-5 (IIEF-5) [14]; (IV) Erection Hardness Score (EHS) [15]; (V) NHANES food frequency questionnaire (FFQ) [16]; (VI) Overall plant-diet index (PDI) and healthful plant-diet index (hPDI). The FFQ, a 154-question survey, was developed and validated by the National Institute of Health, National Cancer Institute [17], and was used to evaluate frequency of food intake over the prior 12 months. To minimize subjective effects on the results, the investigator was blinded to the information except for the scales and each participant finished the questionnaire on their own. Scales to assess psychological disorders and ejaculation function were performed during the population selection and all the participants had normal ejaculation function and psychological status, therefore results on these were not shown. Detailed physical examination included body weight (kg), height (cm), systolic blood pressure (SBP, mmHg) and diastolic blood pressure (DBP, mmHg), waist circumference (WC, cm), and body mass index (BMI, kg/m^2^). Notably, supine WC was measured with slight breath and at the level of the umbilicus.

PDI and hPDI were converted from FFQ data based on a previously validated methodology [7]. PDI differentiates patients based on their frequency of consumption of plant foods. Meanwhile, hPDI could differentiate further by distinguishing healthy plant foods from unhealthy plant foods and animal foods. All the FFQ responses were converted into daily serving sizes and grouped into 17 categories (Supplementary Table 1, in Additional file 1). A score from 1 to 5 was given based on their consumption of foods within each category. When calculating PDI, a score of 5 in plant foods represents the highest quintile of consumption of foods, and a score of 1 is the highest quintile of consumption of animal foods. To calculate hPDI, a score of 5 in healthful plant foods represents the highest quintile of consumption of foods, and a score of 1 is the highest quintile of consumption of both animal foods and unhealthful plant foods.

Blood samples were collected by a trained examiner from 7:30 A.M. to 9:30 A.M. following 12 h of fasting. Blood analysis included total testosterone (TT), free testosterone (FT), and metabolic syndrome-related factors: fasting blood glucose (FBG), triglyceride (TG), total cholesterol (TC), high-density lipoprotein cholesterol (HDL-C), low-density lipoprotein cholesterol (LDL-C), and C-reactive protein (CRP). Additionally, nitric oxide (NO) and E-selectin, two surrogated items of erectile endothelial function, were measured using Enzyme-Linked Immunosorbent Assay (ELISA) procedures in validated assay kits.

### Diagnostic criteria

The Chinese version of IIEF-5 is a five-item questionnaire, which assesses erectile function, intercourse satisfaction, orgasmic function, sexual desire, and overall satisfaction in the past 6 months. Based on the score, ED could be classified into normal mild ED (score 21–17), mild to moderate ED (score 16–12), moderate ED (score 11–8), and severe ED (score 7–1). NPTR monitoring was another measurement of EF. We used the criterion raised by Hatzichristou et al. that a functional erectile was an erectile event of at least 60% rigidity recorded on the tip of the penis and lasted for > 10 min [18]. Organic ED patients were those who had IIEF-5 scores less than 22 and failed to meet the NPTR criterion.

Metabolic syndrome (MetS) was diagnosed according to the criteria of the Harmonization 2009 definition for Asians, which defined the MetS as the simultaneous presence of any 3 of the followings: abdominal obesity (WC not less than 90 cm), BP ≥130/85 mmHg or current use of antihypertensive medication, FBG > 100 mg/dL or current use of oral diabetes medication or insulin, TG > 150 mg/dL or drug therapy, and HDL-C < 40 mg/dL or drug therapy (Supplementary Table 2, in Additional file 1).

### Data analysis

PASS software version 15.0 was used to calculate the sample size. The results revealed that to achieve 90.00% power when the population effect size is 0.50 and the significance level (alpha) is 0.050 using a two-sided two-sample equal-variance t-test, group sample sizes of 86 and 86 are essential (totally 172 participants). We analyzed data from the study by using SPSS version 26.0 (SPSS Inc., Chicago, IL, USA). All the qualitative data were presented as frequency (proportions). The quantitative data were shown in mean ± standard deviation (SD) or median (interquartile range, IQR). *χ*^*2*^ test was used to compare categorical data and continuous variables were compared by independent student’s *t*-test for normally distributed data or Mann–Whitney *U* test for nonparametric data. Spearman correlation test was performed to determine the correlation between the food subgroups and IIEF-5 scores. We conducted univariate logistic analysis (UVA) and multivariate logistic analysis (MVA) to analyze associations between ED and related factors. Unadjusted, partly adjusted, and additionally adjusted MVA was performed to investigate the relationships between PDI and ED severity and the presence of ED, and also the links between hPDI and ED severity and the presence of ED. Trend analysis was also done. A two-tailed *P* < 0.05 indicated statistical significance in all data analyses.

## Results

The basic characteristics of the 184 participants were presented in Table 1. All the participants were Chinese and the majority of the subjects was employed and urban resident. There were no significant differences between the two groups in terms of age, partner age, duration of the relationship, frequency of intercourse, residence, occupation, education level, and also monthly income (*P* > 0.05 for all). Significant differences could only be observed between the two groups in lifestyle, including smoking, exercise, and alcohol intake (*P* < 0.05 for all).

**Table 1:** Basic features of men included in the study

	ED (*n* = 92)		Control (*n* = 92)		*P*-value
	n	%	n	%	
Age, years	44.4 ± 13.7		45.9 ± 12.6		0.441
Partner age, years	41.8 ± 10.5		41.4 ± 10.1		0.793
Duration of partnership, years	22.5 ± 11.2		22.8 ± 10.9		0.854
Frequency of intercourse, per month	5.3 ± 3.6		6.0 ± 4.2		0.226
Residence					0.461
Urban	49	53.3	44	47.8	
Rural	43	46.7	48	52.2	
Occupational status					0.879
Unemployed	5	5.4	6	6.6	
Employed	84	91.3	82	89.1	
Retired	3	3.3	4	4.3	
Educational status					0.991
No compulsory education	5	5.4	5	5.4	
Compulsory education	11	12.0	12	13.0	
High school	55	59.8	53	57.6	
Higher education	21	22.8	22	24.0	
Monthly income, CNY	3962.8 ± 574.3		3876.2 ± 545.8		0.296
Lifestyle (current)					
Smoking (Yes)^a^	35	38.0	21	22.8	**0.025**
Exercise (Yes)^b^	29	31.5	43	46.7	**0.034**
Alcohol (Yes)^c^	26	28.3	12	13.0	**0.011**

*Abbreviations*: *CNY* China Yuan

^a^ Current or former smoker

^b^ Physical activity ≥moderate

^c^ At least one drink per week

**Fig. 2:**
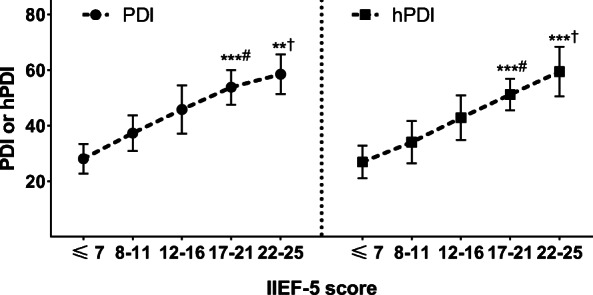
The trend analysis of associations between IIEF-5 scores and PDI or hPDI. ^#^ Trend analysis within ED group. ^†^ Comparison of PDI or hPDI by Student *t*-test between the control group (IIEF-5 score: 22–25) and mild ED group (IIEF-5 score: 17–21). ** < 0.01, *** < 0.001. Abbreviations: hPDI: healthful plant-based diet index; IIEF-5: International Index of Erectile Function-5; PDI: overall plant-based diet index

Table 2 showed comparisons of the outcomes of questionnaires, physical examination, and blood tests between the two groups. Briefly, IIEF-5 and EHS score, two major ED measurements in the study were significantly different between the ED group and control group (*P* < 0.05). Furthermore, the PDI and hPDI in the ED group were significantly lower than the control group (*P* < 0.05 for both). As for overall correlation between consumption of each food subgroup and erectile function (IIEF-5 score), no significant results were observed in all these categories (*P* > 0.05 for all) (Supplementary Table 3, in Additional file 1). The results of UVA showed that the presence of ED was significantly associated with BMI, MetS, NO and E-selectin levels, PDI, and hPDI (all *P* < 0.05), but not TT levels (*P* = 0.135). Consistent results were also observed in multivariate analysis (Table 3). Notably, results from MVA indicated that both the PDI and hPDI increased significantly as the IIEF-5 scores increased within the ED group (Fig. 2 and Table 4). Additionally, multi-model MVA was done as the sensitivity analysis, results of which presented that the results of the associations between diet (PDI or hPDI) and ED severity and the presence of ED were robust (Supplementary Tables 4–7, in Additional file 1).

**Table 2:** Main outcomes of scales and examinations

	ED (*n* = 92)	Control (*n* = 92)	*P*-value
TT, ng/mL	4.4 ± 2.3	4.2 ± 2.0	0.530
FT, pg/mL	86.3 ± 14.7	88.4 ± 16.7	0.366
IIEF-5 score	13.3 ± 6.8	23.3 ± 1.5	**0.000**
EHS score^#^	2 (1, 3)	3 (3, 4)	**0.022**
Weight, kg	78.2 ± 14.5	74.5 ± 15.6	0.097
Height, cm	174.7 ± 8.2	175.0 ± 7.4	0.795
BMI, kg/m^2^	25.6 ± 5.9	24.3 ± 6.5	0.157
Blood pressure, mmHg			
Systolic	132.4 ± 20.2	134.0 ± 21.3	0.602
Diastolic	83.6 ± 14.1	85.2 ± 12.9	0.423
Waist circumference, cm	89.2 ± 9.6	86.3 ± 9.1	**0.037**
Other blood measurements			
FBG, mmol/L	6.4 ± 1.7	5.7 ± 1.3	**0.002**
TG, mmol/L	1.7 ± 0.8	1.8 ± 0.7	0.368
TC, mmol/L	5.2 ± 1.3	5.0 ± 1.4	0.317
HDL-C, mmol/L	1.6 ± 0.6	1.5 ± 0.6	0.260
LDL-C, mmol/L	3.1 ± 0.8	3.0 ± 0.9	0.427
NO, μmol/L	30.8 ± 21.3	42.4 ± 20.2	**0.000**
E-selectin, ng/mL	37.3 ± 12.7	12.9 ± 4.9	**0.000**
CRP, mg/L	4.9 ± 3.2	3.9 ± 3.0	**0.030**
MetS^m^	36 (39.1%)	20 (21.7%)	**0.010**
PDI^n^	47.1 ± 7.5	58.5 ± 7.1	**0.000**
hPDI^n^	46.8 ± 8.2	59.4 ± 8.9	**0.000**

*Abbreviations*: *BMI* body mass index, *CRP* C-reactive protein, *ED* erectile dysfunction, *EHS* Erection Hardness Score, *FBG* fasting blood glucose, *FT* free testosterone; *HDL-C* high-density lipoprotein cholesterol, *hPDI* healthful plant-based diet index, *IIEF-5* International Index of Erectile Function-5, *LDL-C* low-density lipoprotein cholesterol, *MetS* metabolic syndrome, *NO* nitric oxide, *PDI* overall plant-based diet index, *TC* total cholesterol, *TG* triglyceride, *TT* total testosterone

^#^ Median (interquartile range 25–75)

^m^ MetS was defined following the criterion shown in Supplementary Table 2, in Additional file 1

^n^ PDI and hPDI were converted from FFQ following the method described in the method section

**Table 3:** Univariate analysis and multivariate analysis for presence of ED

Items	UVA			MVA_1_^a,c^			MVA_2_^b,c^		
	OR	95% CI	*P*-value	OR	95% CI	*P*-value	OR	95% CI	*P*-value
TT, ng/mL	0.987	0.968–1.006	0.135	–	–	–	–	–	–
BMI, kg/m^2^	1.173	1.046–1.300	**0.022**	1.201	1.114–1.288	**0.005**	1.221	1.120–1.323	**0.001**
MetS (Yes vs. No) ^d^	1.213	1.197–1.231	**0.005**	1.323	1.265–1.383	**0.001**	1.354	1.272–1.436	**0.002**
NO, μmol/L	0.679	0.603–0.756	**0.027**	0.693	0.611–0.712	**0.035**	0.671	0.626–0.720	**0.007**
E-selectin, ng/mL	2.022	1.895–2.147	**0.000**	1.765	1.631–1.898	**0.000**	1.739	1.682–1.800	**0.002**
Diet (PDI)^e^	0.779	0.721–0.837	**0.001**	0.869	0.795–0.943	**0.004**	–	–	–
Diet (hPDI)^e^	0.602	0.546–0.661	**0.001**	–	–	–	0.784	0.690–0.878	**0.000**

*Abbreviations*: *BMI* body mass index, *CI* confidence interval, *ED* erectile dysfunction, *hPDI* healthful plant-based diet index, *MetS* metabolic syndrome, *MVA* multivariate analysis, *NO* nitric oxide, *OR* odds ratio, *PDI* overall plant-based diet index, *TT* total testosterone, *UVA* univariate analysis

^a^Multivariate analysis included PDI

^b^Multivariate analysis included hPDI

^c^ Adjusting for age, partner age, duration of partnership, frequency of intercourse, residence, occupational and educational status, income, and lifestyle

^d^ MetS was defined following the criterion shown in Supplementary Table 2, in Additional file 1

^e^ PDI and hPDI were converted from FFQ following the method described in the method section

**Table 4:** Multivariate analysis for EF measurements, TT level, BMI, No. of MetS components and PDI or hPDI^a^

	PDI			hPDI		
	β-Coefficient	95% CI	*P*-value	β-Coefficient	95% CI	*P*-value
IIEF-5^b^	0.473	(0.365, 0.580)	**0.022**	0.406	(0.297, 0.515)	**0.004**
NO, μmol/L^b^	0.147	(0.035, 0.236)	**0.005**	0.153	(0.055, 0.250)	**0.002**
E-selectin, ng/mL^b^	−0.129	(−0.256, −0.002)	**0.001**	− 0.140	(− 0.243, − 0.037)	**0.000**
BMI, kg/m^2^	− 0.323	(− 0.425, − 0.220)	**0.006**	−0.372	(− 0.439, − 0.305)	**0.001**
TT, ng/mL^b^	0.109	(−0.084, 0.303)	0.201	0.102	(−0.092, 0.295)	0.514
No. of MetS components ^c^	−2.034	(−2.334, −1.733)	**0.005**	−1.856	(−2.026, − 1.689)	**0.002**

*Abbreviations*: *BMI* body mass index, *EF* erectile function, *hPDI* healthful plant-based diet index, *IIEF-5* International Index of Erectile Function-5, *MetS* metabolic syndrome, *NO* nitric oxide, *PDI* overall plant-based diet index, *TT* total testosterone

β-Coefficient indicates change in items associated with a 1 change in PDI or hPDI

^a^Adjusted for age, residence, occupation, education status, income, lifestyle, and other blood measurements;

^b^Additional adjustment for BMI and No. of MetS components

^c^No. of MetS components corresponded to the number of MetS components in subjects as defined following the criterion shown in Supplementary Table 2, in Additional file 1

## Discussion

In the present study, we studied whether there was an association between plant-based diet and organic ED in a Chinese population. When stratifying plant foods from meat by using the PDI, we found that higher consumption of plant-based diet was related to reduced presence of ED. Consistent findings were also observed when we further stratified healthy plant foods from unhealthy plant foods and meat by using hPDI. Additionally, we found that a lower portion of plant consumption was associated with a lower IIEF-5 score, a lower NO level, and a higher E-selectin level, which indicated a more severe ED.

In the study, mean PDI and hPDI in participants were 52.8 and 53.1, respectively. Results on this were consistent with previous studies investigating Chinese diet composition [19, 20], which indicated the representativeness of the selected sample. Kuchakulla et al. reported the mean PDI and hPDI in American men were 50.4 and 50.8, respectively [21]. The differences on PDI and hPDI might be attributed to diet culture differences between China and America, i.e. Chinese diets are typically lower in daily intakes of dairy. Furthermore, the control group has a significantly higher score of both PDI and hPDI than the ED group, which indicated less consumption of plant-based diet in ED population.

Currently, studies assessing the role of plant-based diet on ED are limited and insufficient. In contrast, lots of researches focused on associations between other diets and erectile function. Evidence showed that men adhering to the Mediterranean diet tended to have a lower rate of ED [22, 23]. Furthermore, some prospective studies and randomized controlled trials (RCTs) indicated that weight loss through low-fat diets in obese ED patients could improve IIEF-5 scores [24, 25]. Other diet compositions, like organic diet and ingredient supplement diet, like nuts, were also reported related to improved IIEF-5 scores [3, 26]. When it comes to plant-based diet, a previous study conducted by Liu et al. indicated that adequate fruit and vegetable intakes were not associated with improved ED [27]. However, two concerns were mentioned by the authors. Firstly, their population mainly consisted of Chinese elderly men, who were not sexually active within previous 6 months. Secondly, they did not adjust for comorbidities, such as hypertension, dyslipidemia, and diabetes in the statistical calculation. Another study without the two limitations demonstrated a protective role of fruit and vegetable consumption against ED in Canadian diabetic population [28]. In the study, we used a graded method, PDI and hPDI, to measure the plant-based diet consumption in a selected population, collected MetS components from all the participants, introduced surrogated items of erectile endothelial assessment, and drew conclusions based on MVA. Briefly, our results supported the protective effect of plant-based diet against ED. However, further well-designed prospective and RCTs are warranted to give a solid conclusion.

Our findings may have some practical implications. Firstly, we conducted a clinical study to investigate the relationship between plant-based diet and ED in China. However, the mechanisms behind the association should be explored in experimental studies, especially the role of anti-inflammatory and antioxidant effects of dietary fibers and polyphenols in penile erection [8]. Secondly, the findings can be used in patient counseling. Men can commit to plant-based diets to lose weight or control weight and they did not need to worry about affecting their TT level. Moreover, more plant-based diet intakes may lower the possibility of getting ED as well as the severity of ED within ED population. It is especially important for those who want to lose weight, stay healthy, and reduce risks of developing several chronic non-communicable diseases, such as type 2 diabetes, malignancy, and coronary heart disease etc. [29]. Notably, to select an appropriate diet for individuals, we should take personal preferences, regional differences, diet habits, and nutrition rationality into consideration. Thirdly, much evidence has indicated that high-income society tended to lower the environmental footprint from food production through sustainable diets [30]. Reduction in animal-based food consumption is proved to reduce greenhouse gas emission and water consumption [9]. Thus, the plant-based diet has a positive impact on environment and also is not harmful to human health, which is conducive for governments to make policies to advocate diet modification. However, it should be noted that men are always recommended to have a diverse diet and any excessive diet will result in adverse consequences. Additionally, further studies are required to validate our findings before their applications.

In the study, controls were selected from a nonfunctional adrenal adenoma follow-up cohort from the two medical centers. Patients with incidentally detected small adrenal adenoma (usually less than 2 cm) will be recommended for follow-ups in the two hospitals. The management of the adrenal incidentaloma was conducted following the guideline made by European Society of Endocrinology [31]. All these patients were consulted in the department of endocrinology in TMUGH and SHTMU and whether the adrenal mass had function was determined by clinical presentations, imaging, and mainly by related experimental tests, such as dexamethasone suppression testing, serum cortisol, 24-h urinary measure of fractionated metanephrines and catecholamines, plasma aldosterone and renin, and sex hormones and steroid precursors. These evaluations were performed every 6 months. Only when it proved that the adenoma was non-functional, will the patient be recorded in the follow-up list and we selected non-ED subjects from the list as the controls. We think this design is rational not only because some studies in the field of andrology undertake a similar selection method [32, 33], but also for the reason that controls in the study were tested strictly to be ED-free subjects before the enrollment. Moreover, there are no literatures showing the link between non-functional adrenal incidentaloma and diet, thus we supposed that currently the control selection was appropriate for the topic. IIEF-5, instead of the International Index for Erectile Function-15 (IIEF-15) was taken as the evaluation tool in the study. The 15-item version was developed by Rosen et al. in 1997, and a 5-item short version followed in 1999 [14, 34]. The IIEF-15 comprises 15 items including 5 domains: erectile function, orgasmic function, sexual desire, intercourse satisfaction, and overall satisfaction. The IIEF-5 consists of 5 items from the IIEF-15, including 4 from the erectile function domain, and 1 from intercourse satisfaction. Thus, IIEF-5 targeted more on EF and IIEF-5 has now been a widely and a more convenient scale used to evaluate EF in outpatient. There are also many studies only using the IIEF-5 to assess EF rather than the IIEF-15 [35]. Finally, it has been reported that some basic features, like age, occupation, and lifestyle associate with ED, therefore we showed these results in the supplementary file (Supplementary Tables 8–9, in Additional file 1). It should be considered carefully because the relatively small sample selected with aim of investigating the relationship between plant-based diet and ED may not reveal the real associations between these adjusted factors and ED.

The main strengths of the study were the introduce of PDI and hPDI to quantitively evaluate the plant-based diet in the population, as well as the use of NO and E-selectin levels as alternative indicators for erectile endothelial function. Our study is also the first to report the association between plant-based diet and ED in China. However, there were some limitations in the study. Firstly, TT levels in men could be fluctuating. Once-tested TT values maybe not the normal status. Secondly, the diet habit was self-reported, recall and self-serving biases may affect the results. Although influence on this might be slight, the findings in the study should be considered tentative until validated by further studies. Thirdly, the study only included cross-sectional data, prospective studies or RCTs are required to provide longitudinal data in plant-based diet and ED to validate our findings. Fourthly, controls and scales selection may be a weakness, as mentioned above, which remains to be justified in the future. Last but not the least, the ED population consisted of organic ED men only, thus findings cannot be applied to other types of ED, such as psychologic ED.

## Conclusions

By conducting the cross-sectional study, we found that more plant-based diet intakes were associated with a reduced presence of ED and less severe ED in China. Plant-based diet could be advocated to keep healthy with benefits for erectile dysfunction. This is the first study to explore the relationship between plant-based diet and erectile dysfunction in China. However, further studies are warranted to validate our findings and reveal the mechanisms.

## Supplementary Information

**Additional file 1: Table S1.** Food items separated into 3 food categories and 17 food groups. **Table S2.** The harmonized diagnostic criteria for metabolic syndrome in Asian. Abbreviations: BP: blood pressure; DM: diabetes mellitus; FBG: fasting blood glucose; HDL: high-density lipoprotein; TG: triglyceride; WC: waist circumference. **Table S3**. Overall consumption of the 17 food group categories and correlation analysis between the food groups and IIEF-5 scores. ^#^ Median (interquartile range 25–75). **Table S4** Multivariate analysis for the presence of ED (not including hPDI). ^a^ Unadjusted MVA. ^b^ Adjusting for age, partner age, duration of partnership, frequency of intercourse, residence, occupational and educational status, and income. ^c^ Adjusting for age, partner age, duration of partnership, frequency of intercourse, residence, occupational and educational status, income, and lifestyle. ^m^ MetS was defined following the criterion shown in Supplementary Table 2, in Additional file 1. ^n^ PDI was converted from FFQ following the method described in the method section. Abbreviations: BMI: body mass index; CI: confidence interval; ED: erectile dysfunction; MetS: metabolic syndrome; MVA: multivariate analysis; NO: nitric oxide; OR: odds ratio; PDI: overall plant-based diet index; TT: total testosterone; UVA: univariate analysis. **Table S5**. Multivariate analysis for the presence of ED (not including PDI). ^a^ Unadjusted MVA. ^b^ Adjusting for age, partner age, duration of partnership, frequency of intercourse, residence, occupational and educational status, and income. ^c^ Adjusting for age, partner age, duration of partnership, frequency of intercourse, residence, occupational and educational status, income, and lifestyle. ^m^ MetS was defined following the criterion shown in Supplementary Table 2, in Additional file 1. ^n^ hPDI was converted from FFQ following the method described in the method section. Abbreviations: BMI: body mass index; CI: confidence interval; ED: erectile dysfunction; hPDI: healthful overall plant-based diet index; MetS: metabolic syndrome; MVA: multivariate analysis; NO: nitric oxide; OR: odds ratio; TT: total testosterone; UVA: univariate analysis. **Table S6**. Multivariate analysis for EF measurements, TT level, BMI, No. of MetS components and PDI. ^†^: Unadjusted MVA; ^*^: Adjusted for age, residence, occupation, education status, income, lifestyle, and MetS related blood measurements; ^#^: Additional adjustment for BMI and No. of MetS components. ^m^: No. of MetS components corresponded to the number of MetS components in subjects as defined following the criterion shown in Supplementary Table 2, in Additional file 1. β-Coefficient indicates change in items associated with a 1 change in PDI. Abbreviations: BMI: body mass index; EF: erectile function; hPDI: healthful plant-based diet index; IIEF-5: International Index of Erectile Function-5; MetS: metabolic syndrome; NO: nitric oxide; PDI: overall plant-based diet index; TT: total testosterone. **Table S7**. Multivariate analysis for EF measurements, TT level, BMI, No. of MetS components and hPDI. ^†^: Unadjusted MVA; ^*^: Adjusted for age, residence, occupation, education status, income, lifestyle, and MetS related blood measurements; ^#^: Additional adjustment for BMI and No. of MetS components. ^m^: No. of MetS components corresponded to the number of MetS components in subjects as defined following the criterion shown in Supplementary Table 2, in Additional file 1. β-Coefficient indicates change in items associated with a 1 change in hPDI. Abbreviations: BMI: body mass index; EF: erectile function; hPDI: healthful plant-based diet index; IIEF-5: International Index of Erectile Function-5; MetS: metabolic syndrome; NO: nitric oxide; PDI: overall plant-based diet index; TT: total testosterone. **Table S8.** Univariate analysis and multivariate analysis for presence of ED, including basic features. ^#^ Unadjusted multivariate analysis included PDI. ^†^ Unadjusted multivariate analysis included hPDI. ^m^ MetS was defined following the criterion shown in Supplementary Table 2, in Additional file 1. ^n^ PDI and hPDI were converted from FFQ following the method described in the method section. Abbreviations: BMI: body mass index; CI: confidence interval; ED: erectile dysfunction; hPDI: healthful plant-based diet index; MetS: metabolic syndrome; MVA: multivariate analysis; NO: nitric oxide; OR: odds ratio; PDI: overall plant-based diet index; TT: total testosterone; UVA: univariate analysis. **Table S9**. Multivariate analysis for EF measurements, TT level, BMI, No. of MetS components and PDI or hPDI^†^. ^†^: Unadjusted MVA; ^m^: No. of MetS components corresponded to the number of MetS components in subjects as defined following the criterion shown in Supplementary Table 2, in Additional file 1. β-Coefficient indicates change in items associated with a 1 change in PDI. Abbreviations: BMI: body mass index; EF: erectile function; hPDI: healthful plant-based diet index; IIEF-5: International Index of Erectile Function-5; MetS: metabolic syndrome; NO: nitric oxide; PDI: overall plant-based diet index; TT: total testosterone.

## Data Availability

The datasets generated during and/or analyzed during the current study are available from the corresponding author on reasonable request.

## Abbreviations

BMI: Body mass index
BP: Blood pressure
CI: Confidence interval
CRP: C-reactive protein
DBP: Diastolic blood pressure
DM: Diabetes mellitus
ED: Erectile dysfunction
EF: Erectile function
EHS: Erection Hardness Score
FBG: Fasting blood glucose
FFQ: Food frequency questionnaire
FT: Free testosterone
HDL-C: High-density lipoprotein cholesterol
hPDI: healthful plant-based diet index
IIEF-5: International Index of Erectile Function-5
IQR: Interquartile range
LDL-C: Low-density lipoprotein cholesterol
LUTS: Lower urinary tract symptoms
MetS: Metabolic syndrome
MVA: Multivariate analysis
NO: Nitric oxide
NPTR: Nocturnal penile tumescence and rigidity
OR: Odds ratio
PDI: Overall plant-based diet index
PE: Premature ejaculation
RCTs: Randomized controlled trials
SBP: Systolic blood pressure
SD: Standard deviation
TC: Total cholesterol
TG: Triglyceride
TT: Total testosterone
UTI: Urinary tract infection
UVA: Univariate analysis
WC: Waist circumference
