# Supplementary material for: The association between plant-based diet and erectile dysfunction in Chinese men
Source: Basic Clin Androl. 2021 May 13;31:11. doi: 10.1186/s12610-021-00129-5 (PMC8117588; doi:10.1186/s12610-021-00129-5)
Supplement: Supplementary file 1 — Additional file 1: Table S1. Food items separated into 3 food categories and 17 food groups. Table S2. The harmonized diagnostic criteria for metabolic syndrome in Asian. Abbreviations: BP: blood pressure; DM: diabetes mellitus; FBG: fasting blood glucose; HDL: high-density lipoprotein; TG: triglyceride; WC: waist circumference. Table S3. Overall consumption of the 17 food group categories and correlation analysis between the food groups and IIEF-5 scores. # Median (interquartile range 25–75). Table S4 Multivariate analysis for the presence of ED (not including hPDI). a Unadjusted MVA. b Adjusting for age, partner age, duration of partnership, frequency of intercourse, residence, occupational and educational status, and income. c Adjusting for age, partner age, duration of partnership, frequency of intercourse, residence, occupational and educational status, income, and lifestyle. m MetS was defined following the criterion shown in Supplementary Table 2, in Additional file 1. n PDI was converted from FFQ following the method described in the method section. Abbreviations: BMI: body mass index; CI: confidence interval; ED: erectile dysfunction; MetS: metabolic syndrome; MVA: multivariate analysis; NO: nitric oxide; OR: odds ratio; PDI: overall plant-based diet index; TT: total testosterone; UVA: univariate analysis. Table S5. Multivariate analysis for the presence of ED (not including PDI). a Unadjusted MVA. b Adjusting for age, partner age, duration of partnership, frequency of intercourse, residence, occupational and educational status, and income. c Adjusting for age, partner age, duration of partnership, frequency of intercourse, residence, occupational and educational status, income, and lifestyle. m MetS was defined following the criterion shown in Supplementary Table 2, in Additional file 1. n hPDI was converted from FFQ following the method described in the method section. Abbreviations: BMI: body mass index; CI: confidence interval; ED: erectile dysfunction; [file 12610_2021_129_MOESM1_ESM.docx]

**Supplemental Table 1.** Food items separated into 3 food categories and 17 food groups

| Category | Groups | Food Items |
| --- | --- | --- |
| **Healthy plant foods** |  |  |
|  | Whole grains | Dark breads and rolls, wheat bread, rye bread, all bran cereal, all-bran extra fiber, |
|  | Fruits | Any fruits |
|  | Vegetables | Carrots, broccoli, Brussel sprouts, cauliflower, tomatoes, spinach, greens, kale, tossed salad, cabbage, coleslaw, hot red chili peppers, other peppers, sweet potatoes, yams, orange squash, butternut, pumpkin, green beans, corn, peas, mushrooms, zucchini, any other vegetables |
|  | Nuts | Peanuts, peanut butter, any other nuts |
|  | Legumes | Kidney beans, pinto beans, refried beans, black beans, baked beans, lentils, chickpeas, tofu |
|  | Tea and coffee | Regular tea, regular coffee |
| **Less health plant foods** |  |  |
|  | Fruit juices | Orange juice, apple juice, grape juice, other fruit juices |
|  | Refined grain | Rice, flour tortillas, white breads, rolls, bagels, English muffins, biscuits, corn bread, muffins, crackers |
|  | Potatoes | White potatoes, French fries, baked and mashed potatoes |
|  | Sugar sweetened and artificially sweetened beverages | Regular colas and sodas, diet colas, diet sodas, Hi-C, Tang, Kool-Aid |
|  | Sweets and desserts | Cakes, cookies, brownies, chocolate, candy, fudge |
| **Animal foods** |  |  |
|  | Animal fat | Butter |
|  | Dairy | Milk (including in cereal), yogurt, ice cream, milkshakes, cheese (all types) |
|  | Egg | Eggs |
|  | Fish or seafood | Fish, shrimp, clams |
|  | Meat | Bacon, sausage, processed meats, liver and organ meats, beef, pork, ham, chicken, turkey |
|  | Miscellaneous animal foods | Pizza, calzone, lasagna, cheese dishes, mayonnaise |

**Supplementary Table 2**. The harmonized diagnostic criteria for metabolic syndrome in Asian.

| Variables | Harmonization |
| --- | --- |
|  | Any 3 of the followings |
| WC | ≥ 90 cm |
| BP or drug therapy | SBP ≥ 130 mm Hg and/or DBP ≥ 85 mm Hg |
| FBG or DM | >100 mg/dL or 5.6 mmol/L |
| TG or drug therapy | >150 mg/dL or 1.70 mmol/L |
| HDL-C or drug therapy | <40 mg/dL or 1.03 mmol/L |

Abbreviations: BP: blood pressure; DM: diabetes mellitus; FBG: fasting blood glucose; HDL: high-density lipoprotein; TG: triglyceride; WC: waist circumference.

**Supplementary Table 3** Overall consumption of the 17 food group categories and correlation analysis between the food groups and IIEF-5 scores.

|  | Overall (n=184) ^#^ | *r* | *P*-value |
| --- | --- | --- | --- |
| Whole grains | 3 (1–4) | 0.103 | 0.231 |
| Fruits | 2 (1–4) | -0.076 | 0.152 |
| Vegetables | 3 (2–4) | -0.054 | 0.175 |
| Nuts | 2 (1–3) | 0.047 | 0.096 |
| Legumes | 2 (1–4) | 0.053 | 0.122 |
| Tea and coffee | 2 (2–4) | 0.118 | 0.068 |
| Fruit juices | 3 (2–4) | -0.083 | 0.249 |
| Refined grain | 3 (2–4) | 0.016 | 0.574 |
| Potatoes | 3 (2–5) | -0.043 | 0.425 |
| Sugar-sweetened beverages | 3 (2–4) | 0.076 | 0.365 |
| Sweets | 3 (2–4) | -0.109 | 0.073 |
| Animal fat | 3 (2–5) | 0.056 | 0.284 |
| Dairy | 3 (2–4) | 0.084 | 0.237 |
| Egg | 3 (2–4) | -0.021 | 0.413 |
| Fish or seafood | 3 (2–4) | 0.036 | 0.656 |
| Meat | 3 (2–4) | -0.022 | 0.462 |
| Miscellaneous | 3 (2–4) | -0.143 | 0.074 |

^#^ Median (interquartile range 25–75)

**Supplementary Table 4** Multivariate analysis for the presence of ED (not including hPDI).

| Items | MVA_1_ ^a^ | | | MVA_2_ ^b^ | | | MVA_3_ ^c^ | | |
| --- | --- | --- | --- | --- | --- | --- | --- | --- | --- |
|  | OR | 95% CI | *P*-value | OR | 95% CI | *P*-value | OR | 95% CI | *P*-value |
| TT, ng/mL | — | — | — | — | — | — | — | — | — |
| BMI, kg/m^2^ | 1.177 | 0.942-1.411 | 0.155 | 1.184 | 1.106-1.264 | **0.014** | 1.201 | 1.114-1.288 | **0.005** |
| MetS (Yes vs. No) ^m^ | 1.252 | 1.204-1.304 | **0.003** | 1.297 | 1.258-1.336 | **0.007** | 1.323 | 1.265-1.383 | **0.001** |
| NO, μmol/L | 0.668 | 0.610-0.727 | **0.001** | 0.684 | 0.609-0.760 | **0.009** | 0.693 | 0.611-0.712 | **0.035** |
| E-selectin, ng/mL | 1.983 | 1.826-2.141 | **0.000** | 1.825 | 1.783-1.867 | **0.002** | 1.765 | 1.631-1.898 | **0.000** |
| Diet (PDI) ^n^ | 0.782 | 0.720-0.845 | **0.029** | 0.822 | 0.760-0.885 | **0.001** | 0.869 | 0.795-0.943 | **0.004** |

^a^ Unadjusted MVA.

^b^ Adjusting for age, partner age, duration of partnership, frequency of intercourse, residence, occupational and educational status, and income.

^c^ Adjusting for age, partner age, duration of partnership, frequency of intercourse, residence, occupational and educational status, income, and lifestyle.

^m^ MetS was defined following the criterion shown in Supplementary Table 2, in Additional file 1.

^n^ PDI was converted from FFQ following the method described in the method section.

Abbreviations: BMI: body mass index; CI: confidence interval; ED: erectile dysfunction; MetS: metabolic syndrome; MVA: multivariate analysis; NO: nitric oxide; OR: odds ratio; PDI: overall plant-based diet index; TT: total testosterone; UVA: univariate analysis.

**Supplementary Table 5** Multivariate analysis for the presence of ED (not including PDI).

| Items | MVA_1_ ^a^ | | | MVA_2_ ^b^ | | | MVA_3_ ^c^ | | |
| --- | --- | --- | --- | --- | --- | --- | --- | --- | --- |
|  | OR | 95% CI | *P*-value | OR | 95% CI | *P*-value | OR | 95% CI | *P*-value |
| TT, ng/mL | — | — | — | — | — | — | — | — | — |
| BMI, kg/m^2^ | 1.182 | 0.950-1.417 | 0.094 | 1.202 | 1.108-1.297 | **0.005** | 1.221 | 1.120-1.323 | **0.001** |
| MetS (Yes vs. No) ^m^ | 1.271 | 1.222-1.323 | **0.011** | 1.308 | 1.273-1.345 | **0.000** | 1.354 | 1.272-1.436 | **0.002** |
| NO, μmol/L | 0.672 | 0.613-0.731 | **0.007** | 0.692 | 0.612-0.772 | **0.004** | 0.671 | 0.626-0.720 | **0.007** |
| E-selectin, ng/mL | 1.974 | 1.828-2.120 | **0.000** | 1.836 | 1.774-1.899 | **0.001** | 1.739 | 1.682-1.800 | **0.002** |
| Diet (hPDI) ^n^ | 0.765 | 0.714-0.816 | **0.002** | 0.818 | 0.756-0.882 | **0.023** | 0.784 | 0.690-0.878 | **0.000** |

^a^ Unadjusted MVA.

^b^ Adjusting for age, partner age, duration of partnership, frequency of intercourse, residence, occupational and educational status, and income.

^c^ Adjusting for age, partner age, duration of partnership, frequency of intercourse, residence, occupational and educational status, income, and lifestyle.

^m^ MetS was defined following the criterion shown in Supplementary Table 2, in Additional file 1.

^n^ hPDI was converted from FFQ following the method described in the method section.

Abbreviations: BMI: body mass index; CI: confidence interval; ED: erectile dysfunction; hPDI: healthful overall plant-based diet index; MetS: metabolic syndrome; MVA: multivariate analysis; NO: nitric oxide; OR: odds ratio; TT: total testosterone; UVA: univariate analysis.

**Supplementary Table 6** Multivariate analysis for EF measurements, TT level, BMI, No. of MetS components and PDI.

|  | Model 1^†^ | | | Model 2^*^ | | | Model 3^#^ | | |
| --- | --- | --- | --- | --- | --- | --- | --- | --- | --- |
|  | β-Coefficient | 95% CI | *P*-value | β-Coefficient | 95% CI | *P*-value | β-Coefficient | 95% CI | *P*-value |
| IIEF-5 | 0.566 | (0.394, 0.736) | **0.039** | 0.492 | (0.385, 0.598) | **0.014** | 0.473 | (0.365, 0.580) | **0.022** |
| NO, μmol/L | 0.159 | (0.030, 0.290) | **0.021** | 0.150 | (0.038, 0.273) | **0.002** | 0.147 | (0.035, 0.236) | **0.005** |
| E-selectin, ng/mL | -0.143 | (-0.227, -0.058) | **0.000** | -0.138 | (-0.234, -0.042) | **0.001** | -0.129 | (-0.256, -0.002) | **0.001** |
| BMI, kg/m^2^ | -0.373 | (-0.445, -0.302) | **0.002** | -0.323 | (-0.425, -0.220) | **0.006** | — | — | — |
| TT, ng/mL | 0.082 | (-0.073, 0.235) | 0.086 | 0.101 | (-0.090, 0.291) | 0.302 | 0.109 | (-0.084, 0.303) | 0.201 |
| No. of MetS components ^m^ | -1.786 | (-2.153, -1.420) | **0.042** | -2.034 | (-2.334, -1.733) | **0.005** | — | — | — |

^†^: Unadjusted MVA;

^*^: Adjusted for age, residence, occupation, education status, income, lifestyle, and MetS related blood measurements;

^#^: Additional adjustment for BMI and No. of MetS components.

^m^: No. of MetS components corresponded to the number of MetS components in subjects as defined following the criterion shown in Supplementary Table 2, in Additional file 1.

β-Coefficient indicates change in items associated with a 1 change in PDI.

Abbreviations: BMI: body mass index; EF: erectile function; hPDI: healthful plant-based diet index; IIEF-5: International Index of Erectile Function-5; MetS: metabolic syndrome; NO: nitric oxide; PDI: overall plant-based diet index; TT: total testosterone.

**Supplementary Table 7** Multivariate analysis for EF measurements, TT level, BMI, No. of MetS components and hPDI.

|  | Model 1^†^ | | | Model 2^*^ | | | Model 3^#^ | | |
| --- | --- | --- | --- | --- | --- | --- | --- | --- | --- |
|  | β-Coefficient | 95% CI | *P*-value | β-Coefficient | 95% CI | *P*-value | β-Coefficient | 95% CI | *P*-value |
| IIEF-5 | 0.491 | (0.312, 0.670) | **0.002** | 0.470 | (0.308, 0.632) | **0.011** | 0.406 | (0.297, 0.515) | **0.004** |
| NO, μmol/L | 0.176 | (0.063, 0.287) | **0.011** | 0.166 | (0.062, 0.269) | **0.007** | 0.153 | (0.055, 0.250) | **0.002** |
| E-selectin, ng/mL | -0.173 | (-0.264, -0.083) | **0.002** | -0.154 | (-0.249, -0.060) | **0.000** | -0.140 | (-0.243, -0.037) | **0.000** |
| BMI, kg/m^2^ | -0.376 | (-0.442, -0.311) | **0.015** | -0.372 | (-0.439, -0.305) | **0.001** | — | — | — |
| TT, ng/mL | 0.125 | (-0.118, 0.369) | 0.392 | 0.107 | (-0.095, 0.310) | 0.147 | 0.102 | (-0.092, 0.295) | 0.514 |
| No. of MetS components ^m^ | -1.973 | (-2.105, -1.840) | **0.034** | -1.856 | (-2.026, -1.689) | **0.002** | — | — | — |

^†^: Unadjusted MVA;

^*^: Adjusted for age, residence, occupation, education status, income, lifestyle, and MetS related blood measurements;

^#^: Additional adjustment for BMI and No. of MetS components.

^m^: No. of MetS components corresponded to the number of MetS components in subjects as defined following the criterion shown in Supplementary Table 2, in Additional file 1.

β-Coefficient indicates change in items associated with a 1 change in hPDI.

Abbreviations: BMI: body mass index; EF: erectile function; hPDI: healthful plant-based diet index; IIEF-5: International Index of Erectile Function-5; MetS: metabolic syndrome; NO: nitric oxide; PDI: overall plant-based diet index; TT: total testosterone.

**Supplementary Table 8** Univariate analysis and multivariate analysis for presence of ED, including basic features.

| Items | UVA | | | MVA_1_^#^ | | | MVA_2_^†^ | | |
| --- | --- | --- | --- | --- | --- | --- | --- | --- | --- |
|  | OR | 95% CI | *P*-value | OR | 95% CI | *P*-value | OR | 95% CI | *P*-value |
| Age, years | 0.932 | 0.864-1.002 | 0.137 | — | — | — | — | — | — |
| Residence (Urban vs. Rural) | 1.101 | 0.936-1.265 | 0.228 | — | — | — | — | — | — |
| Occupation (Employed vs. None) | 1.026 | 0.763-1.290 | 0.074 | — | — | — | — | — | — |
| Education (Higher vs. Others) | 0.959 | 0.603-1.316 | 0.092 | — | — | — | — | — | — |
| Income, CNY | 1.121 | 0.835-1.413 | 0.258 | — | — | — | — | — | — |
| Smoking (Yes vs. No) | 1.675 | 1.208-2.141 | **0.004** | 1.582 | 1.202-1.967 | **0.000** | 1.576 | 1.187-1.965 | **0.000** |
| Exercise (Yes vs. No) | 0.821 | 0.702-0.940 | **0.000** | 0.899 | 0.786-1.012 | 0.054 | 0.854 | 0.736-0.972 | **0.025** |
| Alcohol (Yes vs. No) | 2.016 | 1.538-2.948 | **0.000** | 1.843 | 1.214-2.432 | **0.002** | 1.835 | 1.126-2.543 | **0.000** |
| FBG, mmol/L | 1.244 | 1.078-1.411 | **0.012** | 1.231 | 1.064-1.395 | **0.015** | 1.244 | 1.122-1.368 | **0.003** |
| TG, mmol/L | 0.965 | 0.670-1.301 | 0.531 | — | — | — | — | — | — |
| TC, mmol/L | 1.001 | 0.732-1.273 | 0.097 | — | — | — | — | — | — |
| HDL-C, mmol/L | 1.028 | 0.586-1.472 | 0.156 | — | — | — | — | — | — |
| LDL-C, mmol/L | 1.115 | 0.743-1.486 | 0.348 | — | — | — | — | — | — |
| CRP, mg/L | 1.212 | 1.052-1.376 | **0.036** | 1.198 | 1.001-1.389 | 0.052 | 1.176 | 0.975-1.378 | 0.067 |
| TT, ng/mL | 0.987 | 0.968-1.006 | 0.135 | — | — | — | — | — | — |
| BMI, kg/m^2^ | 1.173 | 1.046-1.300 | **0.022** | 1.175 | 0.940-1.410 | 0.121 | 1.176 | 0.939-1.415 | 0.102 |
| NO, μmol/L | 0.679 | 0.603-0.756 | **0.027** | 0.665 | 0.611-0.731 | **0.000** | 0.684 | 0.618-0.750 | **0.000** |
| E-selectin, ng/mL | 2.022 | 1.895-2.147 | **0.000** | 1.980 | 1.826-2.139 | **0.000** | 1.957 | 1.809-2.106 | **0.000** |
| Diet (PDI)^n^ | 0.779 | 0.721-0.837 | **0.001** | 0.785 | 0.722-0.848 | **0.007** | — | — | — |
| Diet (hPDI)^n^ | 0.602 | 0.546-0.661 | **0.001** | — | — | — | 0.762 | 0.708-0.823 | **0.001** |

^#^ Unadjusted multivariate analysis included PDI.

^†^ Unadjusted multivariate analysis included hPDI.

^m^ MetS was defined following the criterion shown in Supplementary Table 2, in Additional file 1.

^n^ PDI and hPDI were converted from FFQ following the method described in the method section.

Abbreviations: BMI: body mass index; CI: confidence interval; ED: erectile dysfunction; hPDI: healthful plant-based diet index; MetS: metabolic syndrome; MVA: multivariate analysis; NO: nitric oxide; OR: odds ratio; PDI: overall plant-based diet index; TT: total testosterone; UVA: univariate analysis.

**Supplementary Table 9** Multivariate analysis for EF measurements, TT level, BMI, No. of MetS components and PDI or hPDI^†^.

|  | PDI | | | hPDI | | |
| --- | --- | --- | --- | --- | --- | --- |
|  | β-Coefficient | 95% CI | *P*-value | β-Coefficient | 95% CI | *P*-value |
| Age, years | 0.287 | (-0.194, 0.757) | 0.122 | 0.215 | (-0.186, 0.244) | 0.311 |
| Income, CNY | 0.122 | (-0.261, 0.506) | 0.228 | 0.117 | (-0.254, 0.468) | 0.068 |
| FBG, mmol/L | -1.424 | (-1.865, -0.983) | **0.021** | -1.387 | (-1.802, -0.973) | **0.004** |
| TG, mmol/L | -1.263 | (-1.539, -0.987) | **0.001** | -1.226 | (-1.528, -0.926) | **0.001** |
| TC, mmol/L | -1.572 | (-1.953, -1.191) | **0.027** | -1.549 | (-1.930, -1.168) | **0.000** |
| HDL-C, mmol/L | 1.072 | (0.733, 1.410) | **0.006** | 1.029 | (0.826, 1.234) | **0.003** |
| LDL-C, mmol/L | -0.569 | (-0.806, -0.334) | **0.000** | -0.495 | (-0.796, -0.195) | **0.000** |
| CRP, mg/L | -0.658 | (-0.909, -0.408) | **0.031** | -0.664 | (-0.912, -0.415) | **0.023** |
| IIEF-5 | 0.528 | (0.376, 0.738) | **0.042** | 0.487 | (0.313, 0.662) | **0.001** |
| NO, μmol/L | 0.161 | (0.032, 0.295) | **0.001** | 0.171 | (0.060, 0.282) | **0.000** |
| E-selectin, ng/mL | -0.146 | (-0.224, -0.052) | **0.000** | -0.173 | (-0.262, -0.084) | **0.001** |
| BMI, kg/m^2^ | -0.369 | (-0.438, -0.297) | **0.011** | -0.369 | (-0.437, -0.301) | **0.002** |
| TT, ng/mL | 0.092 | (-0.075, 0.241) | 0.097 | 0.126 | (-0.118, 0.367) | 0.155 |
| No. of MetS components ^m^ | -1.785 | (-2.151, -1.423) | **0.001** | -1.802 | (-2.077, -1.527) | **0.029** |

^†^: Unadjusted MVA;

^m^: No. of MetS components corresponded to the number of MetS components in subjects as defined following the criterion shown in Supplementary Table 2, in Additional file 1.

β-Coefficient indicates change in items associated with a 1 change in PDI.

Abbreviations: BMI: body mass index; EF: erectile function; hPDI: healthful plant-based diet index; IIEF-5: International Index of Erectile Function-5; MetS: metabolic syndrome; NO: nitric oxide; PDI: overall plant-based diet index; TT: total testosterone.
